# Contamination and oxidative stress biomarkers in estuarine fish following a mine tailing disaster

**DOI:** 10.7717/peerj.10266

**Published:** 2020-10-28

**Authors:** Fabrício Â. Gabriel, Rachel Ann Hauser-Davis, Lorena Soares, Ana Carolina A. Mazzuco, Rafael Christian Chavez Rocha, Tatiana D. Saint Pierre, Enrico Saggioro, Fabio Verissimo Correia, Tiago O. Ferreira, Angelo F. Bernardino

**Affiliations:** 1 Departamento de Oceanografia e Ecologia, Universidade Federal do Espírito Santo, Vitória, Espírito Santo, Brasil; 2 Instituto Oswaldo Cruz, Fundação Oswaldo Cruz, Rio de Janeiro, Rio de Janeiro, Brasil; 3 Departamento de Ciências Naturais, Universidade Federal do Estado do Rio de Janeiro, Rio de Janeiro, Rio de Janeiro, Brasil; 4 Departamento de Química, Pontifícia Universidade Católica do Rio de Janeiro, Rio de Janeiro, Rio de Janeiro, Brasil; 5 Departamento de Saneamento e Saúde Ambiental, Fundação Oswaldo Cruz, Rio de Janeiro, Rio de Janeiro, Brasil; 6 Departamento de Ciência do Solo, Escola Superior de Agricultura Luiz Queiroz, Universidade de São Paulo, Piracicaba, São Paulo, Brasil

**Keywords:** Rio Doce, Health risk assessment, Metalloproteins, Environmental pollution, Estuaries

## Abstract

**Background:**

The Rio Doce estuary, in Brazil, was impacted by the deposition of iron mine tailings, caused by the collapse of a dam in 2015. Based on published baseline datasets, the estuary has been experiencing chronic trace metal contamination effects since 2017, with potential bioaccumulation in fishes and human health risks. As metal and metalloid concentrations in aquatic ecosystems pose severe threats to the aquatic biota, we hypothesized that the trace metals in estuarine sediments nearly two years after the disaster would lead to bioaccumulation in demersal fishes and result in the biosynthesis of metal-responsive proteins.

**Methods:**

We measured As, Cd, Cr, Cu, Fe, Mn, Pb, Se and Zn concentrations in sediment samples in August 2017 and compared to published baseline levels. Also, trace metals (As, Cd, Cr, Cu, Fe, Hg, Mn, Pb, Se and Zn) and protein (metallothionein and reduced glutathione) concentrations were quantified in the liver and muscle tissues of five fish species (*Cathorops spixii*, *Genidens genidens*, *Eugerres brasilianus*, *Diapterus rhombeus* and *Mugil* sp.) from the estuary, commonly used as food sources by local populations.

**Results:**

Our results revealed high trace metal concentrations in estuarine sediments, when compared to published baseline values for the same estuary. The demersal fish species *C. spixii* and *G. genidens* had the highest concentrations of As, Cr, Mn, Hg, and Se in both, hepatic and muscle, tissues. Trace metal bioaccumulation in fish was correlated with the biosynthesis of metallothionein and reduced glutathione in both, liver and muscle, tissues, suggesting active physiological responses to contamination sources. The trace metal concentrations determined in fish tissues were also present in the estuarine sediments at the time of this study. Some elements had concentrations above the maximum permissible limits for human consumption in fish muscles (e.g., As, Cr, Mn, Se and Zn), suggesting potential human health risks that require further studies. Our study supports the high biogeochemical mobility of toxic elements between sediments and the bottom-dwelling biota in estuarine ecosystems.

## Introduction

Estuaries are among the most threatened coastal ecosystems and are continually impacted by anthropogenic activities, which often increase the input of organic and inorganic pollutants to the water and sediment (Muniz et al., 2006; Hadlich et al., 2018; Lu et al., 2018; Varzim et al., 2019). Pollutants released into estuarine ecosystems include toxic metals and metalloids that are stable, and considered persistent environmental contaminants, that is, they are not biodegraded (Gómez-Parra et al., 2000; Garcia-Ordiales et al., 2018). The released contaminants typically decrease water and sediment quality, with impacts to estuarine biodiversity and productivity (Lotze et al., 2006). Therefore, understanding the fate and ecological risks of pollutants is critical to access environmental risks associated with their presence in coastal and marine ecosystems (Mayer-Pinto, Matias & Coleman, 2016).

In November 2015, the Rio Doce estuary in southeast Brazil, was severely impacted by the collapse of an iron mine tailing dam, located 600 km upstream. It is estimated that 43 million m^3^ of iron mine tailings were released and severely affected riverine and riparian ecosystems along the path to the estuary and Atlantic Ocean (Do Carmo et al., 2017). Although the released tailings at the breached dam were mainly composed of non-toxic minerals (Almeida et al., 2018), the high content of Fe-oxyhydroxides (goethite—FeOOH, hematite—Fe_2_O_3_) may have promoted chemical binding of metals and metalloids accumulated within the basin during decades of human impacts. As a result, the tailings exhibit high potential for element mobility, especially for Al, As, Ba, Fe, Mn, Pb and Sr, which may be potentially bound to Fe oxides (Segura et al., 2016). Once the tailings reached the estuary, in November 2015, fine sediments containing high concentrations of some elements were immediately deposited on the bottom, raising ecological concerns regarding the long term risks to the estuarine and coastal ecosystems (De Oliveira Gomes et al., 2017; Queiroz et al., 2018; Richard et al., 2020). The tailings initially impacted benthic and fish assemblages in the Rio Doce estuary (De Oliveira Gomes et al., 2017; Andrades et al., 2020). In 2017, nearly two years after the initial impact, chronic effects from trace metal contamination on benthic assemblages were evident (Bernardino et al., 2019). Large quantities of these tailings are currently deposited along upriver floodplains and river banks, which could be continually transported to the estuary and sustain high levels of contaminants. Given the potential mobility of elements between estuarine sediments and organisms (Queiroz et al., 2018), the extent of the trace metal contamination and the effects to fisheries in the estuary are still unclear.

Chemical elements often bioaccumulate in aquatic organisms, causing a range of sub-lethal effects, such as metabolism depression, diseases, and genotoxic damage (Goyer & Clarkson, 1996; Riba et al., 2005). As a result, fishes and other fishery sources may become bioindicators of metal contamination and be used as a proxy for human health risks (Carrola et al., 2014; Lavradas et al., 2014; Ahmed et al., 2015; Gusso-Choueri et al., 2016; Gu et al., 2018). Contamination effects in fishes can be immediately detected by a range of biochemical indicators (or biomarkers), which are widely applied in environmental monitoring programs (Van Der Oost, Beyer & Vermeulen, 2003; Hauser-Davis, Campos & Ziolli, 2012). Certain proteins are specific biomarkers for metal contamination in fish, which are synthesized to act in detoxification mechanisms (Atli & Canli, 2008). These biomarkers may aid in metal sequestration in tissues, subsequent detoxification, and act against oxidative stress (Forman, Zhang & Rinna, 2009; Ruttkay-Nedecky et al., 2013). Metallothioneins (MTs) are low molecular weight proteins that act in the homeostasis of essential trace elements (e.g., Cu and Zn) and in detoxification processes (e.g., As, Cd, Pb, Hg, among others; Hauser-Davis et al., 2014; Kehring et al., 2016). Metallothionein expression increases above certain trace metal contamination thresholds, as the presence of the thiol groups in cysteine residues allows MTs to bind to specific elements. This process protects the organism from trace metal toxicity through immobilization, metabolic unavailability, and subsequent detoxification, occurring mainly in the liver or organs with equivalent function (Kehring et al., 2016; Okay et al., 2016; Pacheco et al., 2017; Van Ael, Blust & Bervoets, 2017). As a result, liver tissues may exhibit high levels of elements when MTs are not efficient at detoxifying these contaminants. Another biomarker, the tripeptide reduced glutathione (GSH, γ-L-glutamyl-L-cysteinyl-glycine), is an important intracellular antioxidant and defense mechanism, which intervenes against intracellular oxidative stress-induced toxicity (Lavradas et al., 2014; Kehring et al., 2016). The sulfhydryl group (–SH), present in cysteine, is involved in protective glutathione functions (reduction and conjugation reactions; Meister, 1992), and protect cells against heavy metal ions (Singhal, Anderson & Meister, 1987).

Previous studies have showed synthesis of MTs and GSH in response to exposure of an aquatic animal to toxic elements (Lavradas et al., 2014; Souza et al., 2018). Biomarker synthesis was observed in liver, muscle, and kidney tissues that work on the capture, storage, and excretion of toxic elements in a variety of aquatic organisms including mussels, crabs, fish, and marine mammals (Lavradas et al., 2014, 2016; Souza et al., 2018; Monteiro et al., 2019). It is noteworthy that similar effects were observed in contaminated freshwater ecosystems in the Rio Doce basin following the Samarco disaster, with fish exhibiting an induction of the proteins and enzymes expression related to contamination and hepatic damage (Weber et al., 2020).

Fish is an important food source for human communities on the coast. The determination of element concentrations in fish species is crucial to improve food quality assessments, and to prevent ingestion of potentially harmful food items by vulnerable local communities (Hauser-Davis et al., 2016; Van Ael, Blust & Bervoets, 2017; Coimbra et al., 2018). Consumption of fish liver, including from *Mugil* sp., is considered a delicacy in some traditional communities (Hauser-Davis et al., 2016), and could highly increase contamination effects through human consumption. Demersal fishes in particular, typically used as food items by Rio Doce communities, can indicate the presence of bioavailable metals in the environment, because they are in close contact with the bottom sediments and accumulated tailings, including associated elements, and may be a key to evaluating potential threats to humans.

Given the potential of chronic bioaccumulation effects from trace metals in the Rio Doce estuary, this study has quantified trace metal contamination and the expression of two detoxification proteins on fish captured nearly two years after the tailings arrival. Our aims were: (i) to determine the trace metal concentrations in sediments, and compare them with published pre-impact reference values and with international sediment quality guidelines (SQGs); (ii) to quantify trace metal contents in the muscle and liver tissues of demersal fish species, and to compare these values with maximum residue levels (MRL) in food items; and (iii) to determine concentrations of oxidative defense and metal detoxification biomarkers in the muscle and liver tissue of five fish species to reveal active physiological responses to trace metal contamination in fish. Our hypothesis was that chronic exposure to contaminated sediments, for 1.7 years following the disaster, would lead to the assimilation of trace metals and to the expression of oxidative defenses in fish. It is well known that metal accumulation in organisms is generally higher in the liver, as it is one of the most important organs for metal detoxification (Hauser-Davis et al., 2016). The metal accumulation in muscle tissues may thus indicate high levels of environmental contamination, suggesting that the liver’s capacity for excretion was exceeded, resulting in bioaccumulation into muscle tissues. Additionally, we have compared metal and metalloid concentrations in fish to reference values from other polluted and pristine estuaries, as well as to Brazilian and international guidelines. This study provides a timely and critical assessment of bioaccumulation in fish that are used for the subsistence of villagers that rely on fisheries from the estuary, and highlights the cumulative consequences of mine tailings to aquatic systems and humans.

## Materials and Methods

### Study area and sampling

The Rio Doce estuary is located in the Eastern Brazil Marine Ecoregion (19° 38′–19° 45′ S and 39° 45′–39° 55′ W). The area has two well-defined seasons, a dry winter (April to September) and a rainy summer (October to March), with an average monthly rainfall of 145 mm and temperature of 25 °C (Bernardino et al., 2015; Bissoli & Bernardino, 2018). The estuary is characterized by a main channel with sand pockets that form at low tide, with water salinity ranging from 0 to 5 (De Oliveira Gomes et al., 2017). The estuary is used by local villagers as a source of subsistence through fisheries and tourism, and estuarine fishes (*Mugil* sp. and *Eugerres brasilianus*) are important food sources (Pinheiro & Joyeux, 2007).

**Figure 1:**
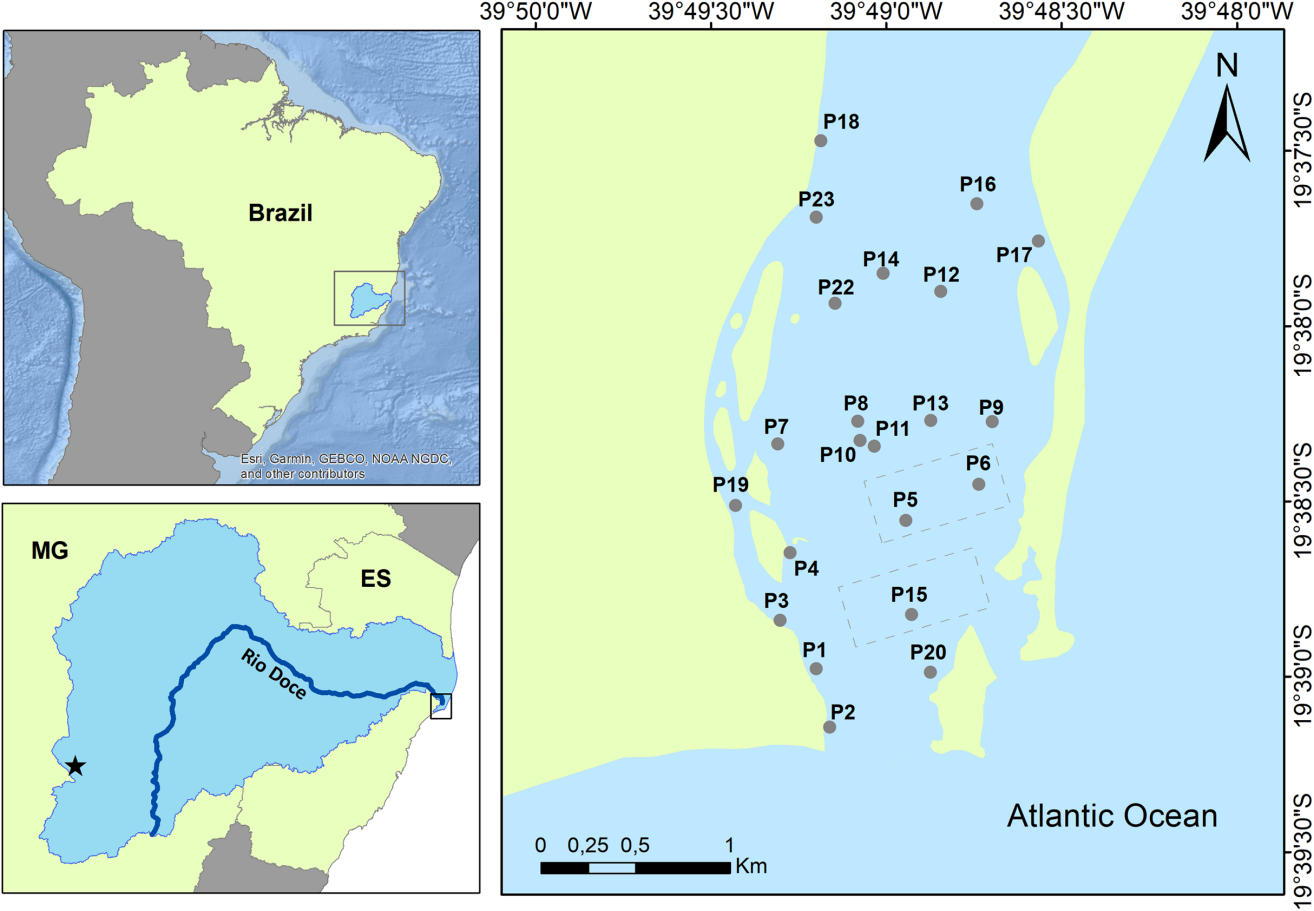
Map of the sampling stations in the Rio Doce estuary, Brazil in August 2017. Fundão dam failure area (star), sediment sampling (circles), and fish sampling areas (rectangle).

The great magnitude of the disaster made it practically impossible to establish control sampling sites, because the entire estuary was highly impacted by the mining tailings. However, evidence indicates increased trace metal contamination in the estuarine sediments, associated with the tailings arrival in November 2015 and subsequent negative effects on both benthic and fish assemblages (De Oliveira Gomes et al., 2017; Queiroz et al., 2018; Andrades et al., 2020; Richard et al., 2020). In August 2017, sediment trace metal concentrations were still significantly above baseline levels in the estuary, with measurable effects on benthic assemblages (Bernardino et al., 2019). Herein, we used the same sediment sampling stations as Bernardino et al. (2019) to determine estuarine contamination nearly two years after the disaster, with additional fish sampling in common fishing areas in the estuary to evaluate potential bioaccumulation effects (Fig. 1).

Qualitative fish sampling was conducted at two sites in the estuary, using a gillnet (5 cm internodes) submerged for 12 h. Estuarine *Cathorops spixii* (*n* = 15), *Genidens genidens* (*n* = 18), *Eugerres brasilianus* (*n* = 18), *Diapterus rhombeus* (*n* = 9), and *Mugil* sp. (*n* = 11) specimens were captured, cryoanesthetized, and stored at 4 °C until laboratory processing. After dissection, fish muscle and liver tissues were separated and stored at −80 °C until analysis. All sampled fish were adults (Table S1 in Supplemental Material) and contained active gonadal maturation, both spawning and post-spawning individuals. All fish included in this study are demersal species with predominant benthic foraging and are typical food items for local villagers (Andrades et al., 2020). Field campaigns were conducted under Sisbio/ICMBio license number 57819-4, with assistance from local villagers.

Surface sediment (0–5 cm) was sampled at 17 random stations along the Rio Doce estuary using a Van-Veen Grab (Fig. 1) and stored in previously decontaminated (30% HNO_3_) containers for processing. These sampling sites are part of a long-term (4-year) monitoring of pre- and post-impacts in the Rio Doce estuary, following the disaster in 2015 (De Oliveira Gomes et al., 2017; Bernardino et al., 2019; Gabriel et al., 2020).

### Sediment analysis

Trace metals were determined in the estuarine sediment samples by tri-acid digestion, using HNO_3_, HF and H_3_BO_3,_ in a microwave oven, according to the EPA 3052 method (United States Environmental Protection Agency (EPA), 1996). The analysis included two-gram aliquots (wet weight) of sediment. Digestion was performed using nine mL of HNO_3_, three mL of HF (1 mol L^−1^) and 5 mL of H_3_BO_3_ (5%). Vessels containing the subsamples were shaken and heated at 110 °C for 4 hours. Subsequently, samples were diluted to 40 mL with deionized water. Finally, 0.1 mL aliquots were analyzed on an ICP-OES spectrometer (iCAP 6200; Thermo Scientific, Waltham, MA, USA). The analyses were performed in triplicate. To guarantee quality control, standard solutions were prepared from dilution of certified standard solutions and certified reference materials (NIST SRM 2709a) and used for comparison to measured and certified values (Table S2). Trace metal concentrations in sediments were then compared to baseline values for the estuary (De Oliveira Gomes et al., 2017) and to sediment quality guidelines (Table 1). The determined metals and metalloid (As, Cd, Cr, Cu, Mn, Pb, Se and Zn) were selected due to their significant increase in concentration post-disaster (Queiroz et al., 2018) and their relevance from a toxicological and biogeochemical perspective. In addition, metal concentrations were compared to local reference values calculated from pre-impact assessment (between 11 and 2 days before the arrival of tailings; De Oliveira Gomes et al., 2017) and sediment quality guidelines (SQGs) for the protection of aquatic life as determined by the National Oceanic and Atmospheric Administration (NOAA as Threshold Effect Level—TEL (limit under which no adverse effects on the biological community is observed) and Probable Effect Level—PEL (probable level where adverse effects in the biological community would occur, Threshold effect concentrations—TEC and Probable effect concentrations—PEC (NOAA, 2008).

**Table 1:** Comparison between mean elements values in sediments with the local reference values (LRV) and sediment quality guidelines (SQGs).

Element	This study		LRV	Sediment quality guidelines (SQGs)			
	Min–max	Mean ± SD		TEL	PEL	TEC	PEC
Cr	18–71.1	47.4 ± 15.2	3.6	52.3	160	43	110
Zn	18.2–78.1	38.7 ± 14.5	1.6	124	271	120	460
Mn	148.7–1,002.7	540.8 ± 220	231	–	–	460	1,100
As	<LQ–28.8	7.8 ± 7.7	3.3	7.24	41.6	9.8	33
Cu	3–15.0	9.4 ± 4.0	1.3	18.7	108	32	150
Pb	5.6–192.9	100.2 ± 47.5	4.7	30.2	112	36	130
Cd	0.6–7.1	3.6 ± 1.4	0.01	7.24	41.6	0.99	5
Se	<LQ–13.6	5.9 ± 4.3	1.0	–	–	–	–

**Note:**

Threshold effect level (TEL), Probable effect level (PEL), Threshold effect concentrations (TEC) and Probable effect concentrations (PEC). All values are reported as mg kg^−1^. Local reference values were calculated from pre-impact assessment in the Rio Doce estuary by De Oliveira Gomes et al. (2017). LOQ for Se and As = 0.01 mg kg^−1^.

### Metals and metalloid in fish

Approximately 100 mg of wet sample (muscle and liver) were weighed in sterile polypropylene tubes, followed by the addition of 1.0 mL of bidistilled HNO_3_. Method accuracy was established by the parallel analyses of procedural blanks (containing only 1.0 mL of bidistilled HNO_3_) and the certified reference material (CRM, DORM-4, dogfish muscle tissue, National Research Council of Canada, Canada), in triplicate. Observed and certified values (mg kg^−1^) for the DORM-4 certified reference material and recovery efficiencies (%) for each element and their respective LOQ (mg kg^−1^) were determined (Table S3). Recovery values were considered adequate for this method, according to Eurachem standards (Eurachem, 1998; Ishak et al., 2015). The samples, blanks and CRM were left for approximately 12 hours overnight, then heated the following morning on a digester block for 4 h at approximately 100 °C. The closed vessels were monitored hourly with manual pressure relief, as necessary. After heating, the samples, CRM and blanks were left to cool at room temperature and made up to appropriate volumes with ultra-pure water (resistivity> 18 MΩ cm). Element quantification was performed by ICP-MS using an ELAN DRC II ICP-MS (Perkin-Elmer Sciex, Norwalk, CT, USA). ^103^Rh was used as the internal standard at 20 µg L^−1^.

### Metallothionein (MT) and reduced glutathione (GSH) in fish

Samples for MT extraction were prepared according to the protocol proposed by Erk et al. (2002). Briefly, muscle and liver samples (50 mg) were homogenized for 3 min in a 300 µL solution with 20 mmol L^−1^ Tris-HCl pH 8.6, phenylmethanesulphonyl fluoride 0.5 mmol L^−1^ as the antiproteolytic agent and β-mercaptoethanol 0.01 % as the reducing agent. The samples were then centrifuged at 20,000 rpm at 4 °C for 60 min. The resulting supernatants were separated from the pellets and placed in new microtubes. Proteins in the samples were denatured by heating the semi-purified supernatants for 10 min at 70 °C, followed by centrifugation for 30 min in the same conditions. Finally, the supernatants containing MT were transferred to new microtubes and frozen at −80 °C until analysis.

Metallothionein quantification via sulfhydryl content determination was performed by UV-Vis spectrophotometry through Ellman’s reaction (Ellman, 1959). The samples were treated with a mixture of 1 mol L^−1^ HCl, 4 mol L^−1^ EDTA and 2 mol L^−1^ NaCl containing 5.5 dithiobis (2-nitrobenzoic acid) buffered in 0.2 mol L^−1^ sodium phosphate, pH 8.0. After incubation for 30 minutes, sample absorbances were determined at 412 nm on a UV-Vis spectrophotometer. Metallothionein concentrations were estimated using an analytical curve plotted with GSH as an external standard and transformed to metallothionein through the known stoichiometric relationship between metallothionein and reduced glutathione, 1:20; GSH contains 1 mole of cysteine per molecule and metallothionein, 20 moles.

The reduced glutathione analysis followed the protocol proposed by Beutler (1975), with modifications introduced by Wilhelm-Filho et al. (2005). Twenty-five milligrams of tissue (liver and muscle) were weighed and homogenized in 350 µL of 0.1 mol L^−1^ sodium phosphate buffer pH 6.5 containing 0.25 mol L^−1^ sucrose. The samples were then centrifuged at 11,000 rpm for 30 minutes at 4 °C. The supernatants were transferred to microtubes and treated with 0.1 mol L^−1^ DTNB at pH 8.0 with a 1:1 ratio. After incubation for 15 minutes in the dark, sample absorbance was determined at 412 nm on a UV-Vis spectrophotometer. Reduced glutathione concentrations were estimated using an analytical curve plotted with GSH as an external standard (Monteiro et al., 2006).

### References for fish consumption

Trace metal levels in fish muscle and liver tissues were compared to maximum permissible levels for consumption, according to the Brazilian Health Regulatory Agency (BRASIL, 1965), the Food and Agriculture Organization of the United Nations (FAO/WHO, 1997), the American Food and Drug Administration (U.S. Food and Drug Administration, 1993), the Environmental Protection Agency (United States Environmental Protection Agency (EPA), 2007), the British Ministry of Forestry, Agriculture and Fisheries (MAFF, 1995), and European Community legislation (European Commission, 2001; Table 2).

**Table 2:** National and international maximum permissible levels (mg kg^−1^) for the ingestion of fish products worldwide.

Agency	Zn	Cu	Cd	Pb	Hg	As	Se	Cr	Mn
ANVISA	50	30	1	2	0.5	1	0.3	0.1	–
FAO/WHO	30	30	1	2	0.5	–	–	–	0.5
US FDA	NA	NA	3.7	–	–	–	–	–	–
US EPA	10–30	1–20	>2	–	–	–	–	–	–
MAFF	50	20	0.2	2	0.3	–	–	–	–
EC	–	–	0.05	0.2	0.5	–	–	–	–

**Note:**

NA = Not Available.

### Statistical analyses

Sediment metal concentrations were averaged across sampling stations and expressed as means (three replicated per site) and standard deviation (SD). Before statistical tests, data distribution was verified by the Shapiro-Wilk test. Because the data was normally distributed, parametric tests were applied. One-way ANOVAs were used to test variations of each element concentration (Zn, Cu, Cd, Pb, Hg, As, Se, Cr and Mn) and biomarkers (MT and GSH) between muscle and liver tissues for each fish species. Pearson’s correlation test was used to verify the existence of significant correlations between trace metal concentrations and metallothionein and reduced glutathione data. A one-way ANOVA test was applied to verify differences between biometric data across fish species. As no statistically significant differences were observed between fish size, weight, and sex, the groups were treated homogeneously without a weight/size stratification range or sex separation.

A Canonical Analysis of Principal Coordinates (CAP; Anderson & Willis, 2003) complemented by multidimensional scaling (Anderson, 2001; McArdle & Anderson, 2001; Oksanen et al., 2018) was performed to evaluate the metal trace metal contamination and stress protein expression. Data was square-root transformed prior to CAP. CAP was then used to identify the metal or group of elements that best explained the variation in stress protein expression among species and to determine the protein that contributed most to the differences among samples. All statistical tests used an α = 0.05 significance level. Graphical and analytical processing was performed in Numbers (Apple Inc.) and R project (R Development Core Team, 2005) using the ‘stats’ and ‘vegan’ packages (Oksanen et al., 2018).

## Results

### Sediment contamination and quality assessments

Overall, the mean As, Cd, Cr, Cu, Mn, Pb, Se and Zn concentrations in sediments were higher than the reference values (pre-impact) for the estuary (De Oliveira Gomes et al., 2017), indicating an accumulation of these elements since 2015 (Table 1). The concentrations of Cd, Cr, Pb, and Zn reached 3.6 ± 1.4 mg kg^−1^, 38.7 ± 14.5 mg kg^−1^, 100.2 ± 47.5 mg kg^−1^ and 47.4 ± 15.2 mg kg^−1^, respectively. These values were 35,900%, 2,319%, 2,031% and 1,217% higher, respectively, than the baseline concentrations reported by De Oliveira Gomes et al. (2017). When compared to sedimentary quality guidelines (NOAA, 2008), sedimentary Pb concentrations were higher than the threshold effect level (TEL) at 94% of the sampled stations (min. 5.6 and max. 192.9 mg kg^−1^). Pb sediment values were also above probable effect level (PEL) at 47% of the sampled stations, above the threshold effect concentrations (TEC) over at 82% of the sampled stations, and above probable effect concentrations (PEC) over at 23% of the sampled sites. Sedimentary Cr and As were higher than the TEL and PEC in 53-23% of the samples. Mn was higher than the TEC in 59% and Cd higher than both the TEC and PEC in 94 and 12% of the samples, respectively. These results suggest that the observed concentrations of As, Cd, Cr, Mn and Pb in the estuarine sediments may cause harmful effects to living organisms. On a toxicity risk scale, the Pb and Cd PECs exceeded sediment concentrations.

### Metal accumulation and biomarkers in fish

We detected higher trace metal concentrations in the liver tissues, when compared to muscle tissues, in fish sampled from the Rio Doce estuary. Zinc concentrations were significantly higher in the liver tissue from all species (ANOVA, DF = 99, *F* = 22.9; *p* < 0.0001), and Mn concentrations were higher only in the liver tissue of *E. brasilianus* (ANOVA, DF = 19, *F* = 30.51; *p* = 0.0309). Cd concentrations were below the limit of quantification (LOQ = 0.0255 mg kg^−1^) in muscle tissues from all species, while Pb displayed the same behavior only in *Mugil* sp. (Table 3).

**Table 3:** Elements concentrations in liver and muscle tissues and percentage (number/total samples) of samples that exceeded maximum permissible levels allowed by Brazilian and international guidelines.

Species	Tissue	As	Cd	Cr	Cu	Hg	Mn	Pb	Se	Zn
*Cathorops spixii* *N* = 15	Muscle	7.46 ± 10.46	<LOQ	0.38 ± 0.14	0.30 ± 0.32	0.22 ± 0.02	0.48 ± 0.97	0.03 ± 0.02	0.53 ± 0.20	8.21 ± 12.60
		(0.17–39.31) 73%	<LOQ 0%	(0.25–0.66) 80%	(0.14–1.27) 13%	(0.02–0.07) 20%	(0.11–3.16) 53%	(0.02–0.07) 0%	(0.29–0.85) 87%	(3.58–46.64) 33%
	Liver	0.31 ± 1.24	0.26 ± 0.17	0.40 ± 0.11	15.13 ± 37.87	0.45 ± 0.52	3.27 ± 1.84	0.18 ± 0.11	4.13 ± 3.42	94.61 ± 203.05^a^
		(0.13–3.64) 40%	(0.16–0.76) 93%	(0.31–0.70) 93%	(0.66–129.10) 93%	(0.03–1.81) 73%	(0.17–5.86) 93%	(0.06–0.47) 13%	(0.34–11.08) 100%	(18.50–693.80) 100%
*Genidens genidens* *N* = 18	Muscle	0.23 ± 4.12	<LOQ	0.34 ± 0.11	0.29 ± -0.13	0.26 ± 0.15	0.43 ± 0.19	0.13 ± 0.07	0.38 ± 0.08	14,00 ± 5.74
		(0.10–15.51) 33%	<LOQ 0%	(0.25–0.58) 100%	(0.11–0.61) 0%	(0.11–0.61) 39%	0.14–0.94 33%	(0.02–0.19) 0%	(0.32–0.61) 100%	(6.61–95.41) 67%
	Liver	0.21 ± 0.35	0.41 ± 0.49	0.41 ± 0.17	8.94 ± 9.48	0.75 ± 0.66	1.49 ± 1.87	0.29 ± 0.40	3.91 ± 1.60	571.43 ± 320.2^a^
		(0.12–1.35) 11%	(0.03–2.36) 94%	(0.30–0.62) 89%	(1.89–46.20) 100%	(0.14–3.02) 89%	(0.89–9.00) 100%	(0.03–1.91) 72%	(2.37–9.75) 100%	(126.00–1310.90) 100%
*Eugerres brasilianus* *N* = 18	Muscle	0.20 ± 0.08	<LOQ	0.39 ± 0.09	0.32 ± 0.40	0.19 ± 0.07	0.35 ± 0.91	0.05 ± 0.08	0.69 ± 0.34	3.22 ± 4.61
		(0.11–0.33) 0%	<LOQ 6%	(0.26–0.51) 44%	(0.13–1.71) 11%	(0.07–0.30) 6%	(0.08–4.22) 17%	(0.02–0.23) 6%	(0.33–1.90) 100%	(1.75–22.97) 6%
	Liver	0.51 ± 0.23	0.19 ± 0.25	0.42 ± 0.11	2.36 ± 0.75	0.11 ± 0.02	7.20 ± 4.10 ^a^	0.05 ± 0.04	2.14 ± 0.84	27.27 ± 25.70^a^
		(0.09–0.91) 0%	(0.04–0.96) 83%	(0.25–0.67) 78%	(0.32–3.66) 94%	(0.06–0.15) 0%	(0.28–17.25) 94%	(0.02–0.14) 0%	(0.58–3.86) 100%	(3.41–119.56) 94%
*Diapterus rhombeus* *N* = 9	Muscle	0.52 ± 0.56	<LOQ	0.21 ± 0.04	0.16 ± 0.03	0.08 ± 0.05	0.30 ± 0.53	0.02 ± 0.01	0.78 ± 0.23	3.81 ± 1.34
		(0.15–1.73) 22%	<LOQ 0%	(0.17–0.27) 100%	(0.14–0.22) 0%	(0.05–0.23) 0%	(0.10–1.67) 33%	(0.02–0.03) 0%	(0.45–1.11) 100%	(2.50–7.10) 0%
	Liver	0.97 ± 0.62	0.05 ± 0.26	0.34 ± 0.11	1.67 ± 0.82	0.10 ± 0.03	9.01 ± 8.29	0.06 ± 0.02	1.26 ± 0.79	32.9 ± 28.68^a^
		(0.26–2.37) 44%	(0.04–0.81) 44%	(0.14–0.58) 100%	(0.16–3.04) 89%	(0.06–0.16) 0%	(0.53–31.90) 100%	(0.03–0.08) 0%	(0.17–2.96) 89%	(1.54–99.79) 89%
*Mugil* sp. *N* = 11	Muscle	0.25 ± 0.53	<LOQ	0.32 ± 0.06	0.30 ± 0.28	0.07 ± 0.06	0.20 ± 0.18	<LOQ	0.50 ± 0.21	3.21 ± 2.74
		(0.17–2.02) 9%	<LOQ 0%	(0.27–0.42) 36%	(0.15–0.73) 9%	(0.05–0.24) 0%	(0.07–0.57) 18%	<LOQ 0%	(0.17–0.97) 82%	(1.81–12.18) 9%
	Liver	1.73 ± 1.99	0.11 ± 0.35	0.46 ± 0.17	47.30 ± 150.60	0.16 ± 0.40	1.85 ± 2.14	0.04 ± 0.30	5.81 ± 9.33	71.71 ± 370.92^a^
		(0.24–7.34) 73%	(0.03–1.17) 82%	(0.30–0.75) 73%	(2.83–539.90) 100%	(0.03–1.23) 36%	(0.93–8.78) 100%	(0.02–0.89) 18%	(1.16–33.38) 100%	(23.80–1351.00) 100%

**Note:**

a LOQ for Cd = 0.0255 and Pb = 0.0126 mg kg^−1^.

**Figure 2:**
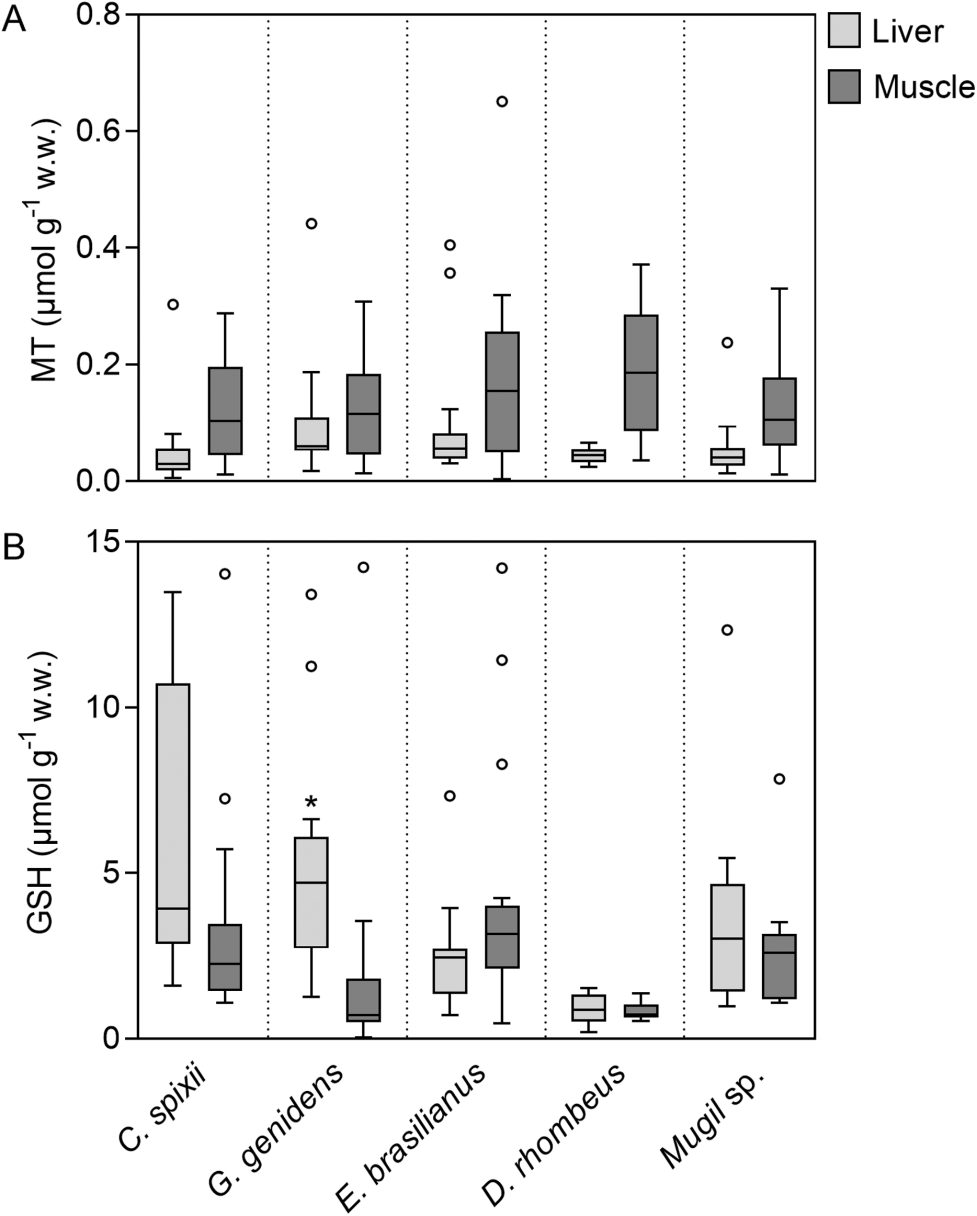
Total metallothionein and reduced glutathione concentrations (μmol g^−1^ wet weight) in estuarine fishes liver and muscle tissues in the Rio Doce estuary. Box plots indicate minimum, maximum, median, quartiles, and outliers (cicle). The asterisk indicates significance at *p* < 0.05.

Contrary to higher metal concentrations (Zn, Mn) in liver tissues, MT levels in the muscle and liver tissues were similar in all sampled fish species (ANOVA, *F* = 2.816; *p* > 0.05; Fig. 2A). In contrast, GSH concentrations were higher in the liver tissue of *C. spixii*, *G. genidens*, *D. rhombeus* and *Mugil* sp. in comparison to their muscle tissue (Fig. 2B), whereas muscle tissues of *E. brasilianus* exhibited higher GSH concentrations (Fig. 2B). A significant difference in GSH levels was observed between liver and muscle tissues for *G. genidens*, with a higher concentration in the liver (ANOVA, *F* = 6.874; *p* < 0.0001; Fig. 2B).

**Figure 3:**
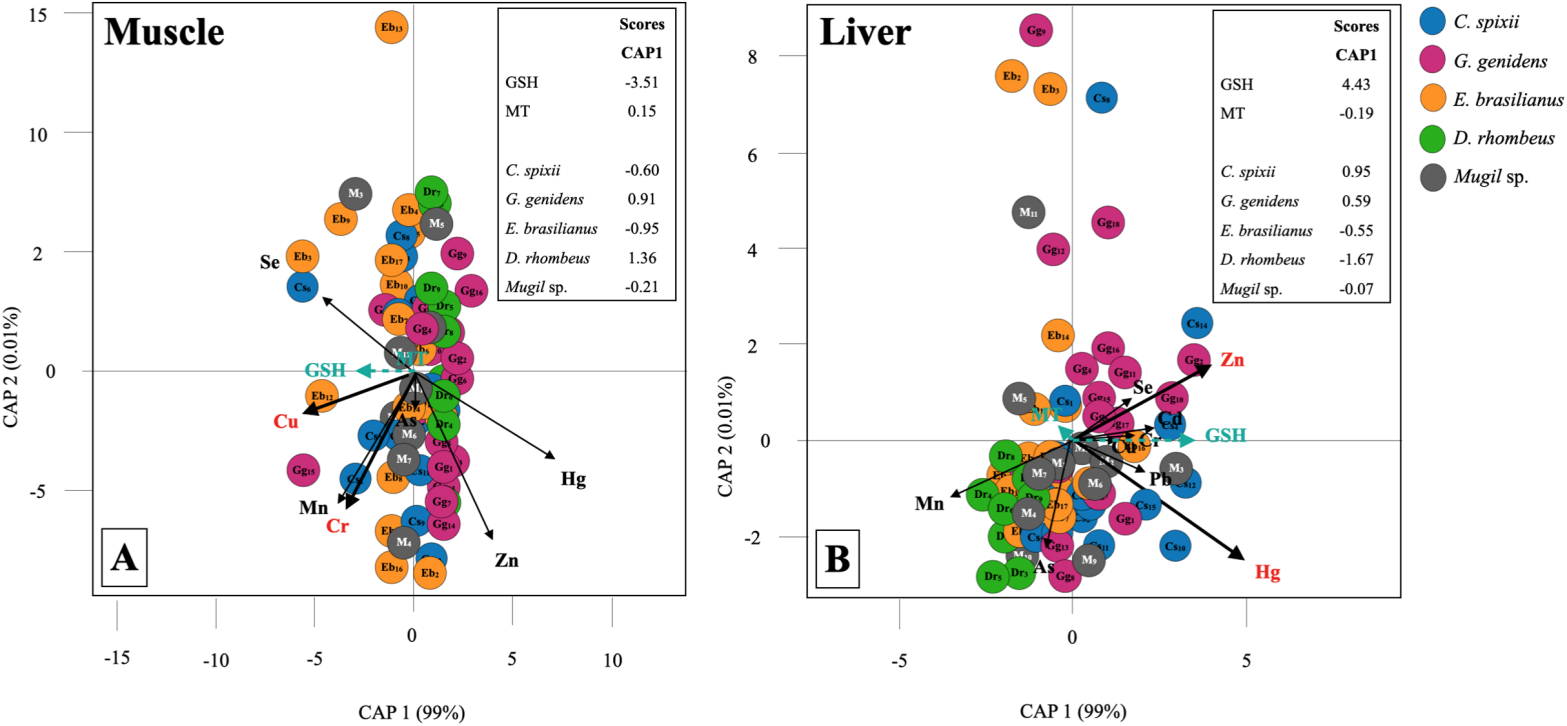
Canonical analysis of principal coordinates (CAP) indicating differences in expression of antioxidant biomarkers and the contribution of elemental contamination in estuarine fishes. Vectors are based on Spearman correlation values > 0.5 (*p* < 0.05) for elements and scores for protein concentration and species (mean score among sampled). The proportions of data explained by axis 1 and 2 are in parentheses.

The CAP analysis indicated a significant association between trace metal concentrations in muscle and liver tissues and the expression of stress proteins in fish (muscle *F* = 2.68, *p* = 0.016, liver *F* = 3.94, *p* = 0.003; Table 4; Fig. 3). In the liver, GSH expression was positively correlated to Zn and Hg concentrations mainly for *C. spixii* and *G. genidens* (Zn *F* = 12.44, *p* = 0.003, Hg *F* = 12.42, *p* = 0.002; Table 4; Fig. 3B). In muscle tissues of *C. spixii* and *E. brasilianus*, Cu and Cr contributed mostlymost to GSH expression (Cu; *F* = 7.12, *p* = 0.012, Cr; *F* = 5.11, *p* = 0.028; Table 4; Fig. 3A). In general, differences in protein expression were the highest in *D. rhombeus* individuals and lowest for *Mugil* sp.

**Table 4:** Results of the canonical analysis of principal coordinates to evaluate the contribution of elements contamination and the variations in the expression of antioxidant biomarkers in estuarine fishes.

	Muscle				Liver			
	CAP 1 (0.99%)	CAP 2 (0.01%)	*F*	*p*	CAP 1 (99%)	CAP 2 (0.01%)	*F*	*p*
Zn	0.28	−0.69	1.61	0.200	0.59	0.22	12.44	**0.003**
Cu	−0.52	−0.14	7.12	**0.012**	0.18	−0.002	1.61	0.203
Cd	–	–	–	–	0.33	0.04	0.29	0.613
Pb	–	–	–	–	0.31	−0.09	2.76	0.097
Hg	0.58	−0.40	3.03	0.084	0.74	−0.37	12.42	**0.002**
As	−0.29	−0.12	0.51	0.490	−0.11	−0.34	2.29	0.153
Se	−0.49	0.31	0.48	0.503	0.24	0.12	0.22	0.631
Cr	−0.43	−0.58	5.11	**0.028**	0.21	0.02	0.14	0.690
Mn	−0.50	−0.56	0.93	0.334	−0.54	−0.18	3.32	0.076

**Note:**

Spearman correlation values for each metal are described for CAP axis 1–2. Note: proportions of variability explained by CAP axes are between parentheses ‘()’, Fisher test statistic, significant results (*p* < 0.05) are in bold. Muscle: *F* = 2.68, *p* = 0.016; Liver: *F* = 3.94, *p* = 0.003.

### Fish contamination and human health

The trace metal concentrations on fish tissues from the Rio Doce estuary were compared to the maximum residue level (MRL) standards. When using the most restrictive guideline for each element, concentrations of As, Cd, Cr, Cu, Hg, Mn, Pb, Se and Zn in fish liver of all species analyzed exceeded the MRL guidelines. The MRL standards were not exceeded for Pb and Hg in *D. rhombeus* and *E. brasilianus* (Table 3). Liver tissue concentrations of Zn, Se and Mn exceeded guidelines for most specimens (>89%). The concentrations of As exceeded the guidelines for *C. spixii* in 73% of the analyzed specimens, and in *G. genidens* for 33% of the muscle tissue samples. Cr concentrations exceeded guidelines in the muscle tissue of all analyzed specimens. Se concentrations also exceeded guidelines in all tissues of *G. genidens* and *E. brasilianus* specimens, in *C. spixii* and *Mugil* sp. liver tissues, and in *D. rhombeus* muscle. Pb and Hg concentrations exceeded guidelines in *C. spixii* and *G. genidens* liver and *E. brasilianus* muscle.

## Discussion

This study revealed a previously unreported bioaccumulation of metals and metalloids in edible fish of the Rio Doce estuary nearly two years after a mine tailing disaster. Trace metal concentrations in fish tissues were associated overall to the expression of biomarkers of oxidative stress in several demersal fish species, suggesting active incorporation of toxic contaminants in the Rio Doce estuary at the time of sampling. Our results support the hypothesis of fish trace metal contamination, which were markedly augmented after the arrival of mine tailings in 2015, and the consequent lower health of aquatic ecosystems in the Rio Doce estuary. Observed increases in the sediment’s trace metal concentrations were above 1000% for most elements studied (Cd, Cr, Pb and Zn), supporting chronic effects of trace metal contamination on the estuarine fauna (Bernardino et al., 2019). The estuarine fish sampled, including *Genidens genidens*, *Diapterus rhombeus* and *Mugil* sp., are typically associated to bottom sediments and often ingest food items buried within sediment matrices (invertebrates; Chaves & Otto, 1998; Chaves & Vendel, 1996; Seixas et al., 2005), which make them especially vulnerable to chemical contamination. Our results suggest that this ingestion may be continuous and active, which is supported by the high trace metal contents in the liver tissues, the primary detoxification organ in fishes (Hauser-Davis, Campos & Ziolli, 2012). In addition, trace metal contamination was observed in bottom-dwelling fish species, typically consumed by local human populations, with potential health implications to villagers that rely on fish as the main protein source for their subsistence. Although baseline trace metal levels are not available for the fish from the estuary, it is very unlikely that the fish sampled 1.7 years after the disaster survived the acute impacts from the tailing in 2015 and would therefore exhibit inherited chemical contamination from the estuary. Hence, our findings suggest a rapid transfer (<2 years) of trace metals and metalloids from sediments to the estuarine biota.

The released mine tailings 600 km upstream of the estuary were initially characterized by low metal concentrations with non-hazardous residues (Almeida et al., 2018). However, the deposited tailings in estuarine soils had significant concentrations of Fe oxides with a high adsorption potential for trace metals that were scavenged during their downstream riverine transport until reaching the estuary (Queiroz et al., 2018). Queiroz et al. (2018) hypothesized that the trace metals bound to Fe oxy-hydroxides would become bioavailable upon Fe reduction in estuarine soils, leading to high ecological risks to the estuarine biota. These risks were evident in the analysis of significant trace metal levels in the estuarine sediments within 2 years after the initial impact (Gabriel et al., 2020). The mobilization of trace metals from tailings have occurred in aquatic riverine ecosystems downstream of the ruptured mining dam (Ferreira et al., 2020; Weber et al., 2020), which supports that, not only the tailings are harmful in aquatic sub-oxic environments, but that toxic trace metals can be adsorbed into these sediments. We detected a relatively rapid bioaccumulation in fish from the Rio Doce estuary, which confirms our previous hypothesis of bioavailability of trace metals that are accumulated in sub-oxic estuarine sediments. Our results reveal that biogeochemical conditions in estuarine sediments may promote bioavailability of trace metals bound to Fe from tailing in the sediments for an extremely long period, as the estuarine ecosystem is a major depocenter of pollutants in coastal zones. If this is confirmed, the fish in the Rio Doce estuary will be contaminated as long as the deposited tailings and associated toxic elements are gradually supplied from upstream transport, and remain deposited on the bottom.

The physiological responses of fish revealed significant correlations of MT and GSH to chemical elements, supporting that the assessed species were under sublethal contamination (or physiological stress) effects at the time of sampling. Although fish sampling can bring stress conditions to fish and lead to biomarker synthesis, the biomarkers (MT and GSH) were significantly correlated to trace metal contamination in both, liver and muscle, tissues. This is also a key advantage of the use of these biomarkers, given that most contaminated areas, including our study, lack baseline metal values in fish. We observed a marked response of biomarker synthesis to Cr, Cu, Hg, and Zn tissue concentrations, which are major contaminants that increased significantly in sediments of the Rio Doce estuary, after the tailings arrival (Queiroz et al., 2018). Therefore, the observed biomarker responses are an additional support for the indication of chronic trace metal contamination effects in this ecosystem, and for fish contamination by bioavailable toxic elements (Gusso-Choueri et al., 2018; Bernardino et al., 2019). The antioxidant function of MTs and GSH and their positive correlations with trace metals proved to be sufficiently sensitive for an impact assessment of the Rio Doce estuary, and thus should be continued during monitoring programs. The trace metal and biomarker concentrations detected in the tissues of these fish seem to reflect the level of contamination of the sediment and its biota.

The general trend of higher MT concentrations in the fish muscle observed in the present study may be associated with trace metal overload in the liver and other excretory organs (e.g., kidneys), with the excess accumulated in the muscle (Pacheco et al., 2017; Souza et al., 2018), suggesting high exposure to the assessed contaminants. However, further monitoring of biomarker expression in fish and trace metal concentrations in fish muscle are required to confirm this hypothesis. GSH expression in liver of *C. spixii*, *E. brasilianus*, *Mugil* sp. and *D. rhombeus* was correlated with Cd, Cr, Hg, Mn and Zn, suggesting that metals create oxidative stress in those individuals (Di Giulio et al., 1995; Atli & Canli, 2008; Monteiro et al., 2008; Sharma & Langer, 2014). Although GSH levels may vary among fish species, the species captured in the Rio Doce estuary exhibited higher GSH expression when compared to fish from uncontaminated freshwater ecosystems upstream (Weber et al., 2020). In addition, fish species from contaminated freshwater ecosystems upstream exhibited similar oxidative stress effects, with higher biomarker levels in addition to internal tissue degeneration (Weber et al., 2020; Macêdo et al., 2020). The GSH expression in fish captured in the estuary suggests that local estuarine ecosystem health has also been severely compromised by trace metal contamination, with possible sub-optimal conditions for the development of fish species (Andrades et al., 2020).

Tissue accumulation of Cd, Cr, Cu, Mn, Pb, Se and Zn was higher in the liver, which is the primary trace metal detoxification organ (Hauser-Davis, Campos & Ziolli, 2012). The liver is rapidly contaminated by toxic metals through the bloodstream, after absorption, so liver trace metal concentrations are assumed to closely resemble those present in the environment (Dural, Göksu & Özak, 2007; De Souza Lima Junior et al., 2002; Bosco-Santos & Luiz-Silva, 2019). Increased trace metal concentrations in muscle tissue from the species *C. spixii* and *G. genidens* may suggest a saturation response for trace metal contamination (Lu et al., 2018; Souza et al., 2018). Several trace metals with high concentrations in fish muscle, including Cu, Zn, Cd and Hg, were also observed at high concentrations in the mine tailings deposited in the estuary (Gabriel et al., 2020). Comparing the concentrations of trace metals in fish from the present study with those found in other studies on the Brazilian coast, those in fish from the Rio Doce were similar or higher than strongly polluted estuaries in Brazil (Table 5). The transfer of bioavailable trace metals from contaminated sediments in coastal ecosystems has been widely reported (Zhu et al., 2015; Hauser-Davis et al., 2016; Gusso-Choueri et al., 2018, Mason et al., 2019), supporting that contaminated sediments in the Rio Doce estuary were the source of the observed trace metals and subsequent physiological effects in fish.

**Table 5:** Elements concentrations (mg kg^−1^) in muscle and liver tissues of fish from the Rio Doce estuary compared with other polluted and pristine estuaries and coastal bays in Brazil.

Species	Location	Tissue	As	Cd	Cr	Cu	Hg	Mn	Pb	Se	Zn	References
*Cathorops spixii*	Cananéia estuary (Near-pristine)	Muscle		0.007		0.067	0.157		0.059		10.95	Azevedo, Hortellani & Sarkis (2012)
	Santos-São Vicente estuary (Polluted)	Muscle		0.005		0.351	0.268		<LOD		10.66	Azevedo, Hortellani & Sarkis (2012)
	Santos-São Vicente estuary (Polluted)	Muscle		0.008		0.494	0.096		0.018		6.30	Azevedo, Hortellani & Sarkis (2012)
	Cananéia estuary (Near-pristine)	Liver					0.25			12.7	1.11	Azevedo et al. (2009)
	Santos Bay (Polluted)	Muscle					0.06					Azevedo et al. (2009)
	Santos Bay (Polluted)	Liver					0.33			15.4	261	Azevedo et al. (2009)
	Paranaguá Bay (Polluted)	Muscle	3.24		0.12	0.22					5.98	Angeli et al. (2013)
	Rio Doce estuary	Muscle	7.46	<LOQ	0.38	0.30	0.22	0.48	0.03	0.53	8.21	This study
	Rio Doce estuary	Liver	0.31	0.26	0.40	15.13	0.45	3.27	0.18	4.13	94.61	This study
*Genidens genidens*	Santos-São Vicente estuary (Polluted)	Muscle		0.007		0.177	0.392		0.01		12.12	Azevedo, Hortellani & Sarkis (2012)
	Santos-São Vicente estuary (Polluted)	Muscle		0.009		0.280	0.105		0.018		9.396	Azevedo, Hortellani & Sarkis (2012)
	Cananéia estuary (Near-pristine)	Muscle		0.006		0.037	0.209		0.057		11.67	Azevedo, Hortellani & Sarkis (2012)
	Paranaguá Bay (Polluted)	Muscle	0.91		0.1	0.22					6.93	Angeli et al. (2013)
	Morrão River estuary (Polluted)	Muscle		0.003		1.29			0.07		80.5	Bosco-Santos & Luiz-Silva (2019)
	Morrão River estuary (Polluted)	Liver		0.24		26.1			7.80		1,201	Bosco-Santos & Luiz-Silva (2019)
	Rio Doce estuary	Muscle	0.23	<LOQ	0.34	0.29	0.26	0.43	0.13	0.38	14.00	This study
	Rio Doce estuary	Liver	0.21	0.41	0.41	8.94	0.75	1.49	0.29	3.91	571.43	This study
*Eugerres brasilianus*	Babitonga Bay (Polluted)	Muscle			0.06						9.28	Bonatti et al. (2004)
	Rio Doce estuary	Muscle	0.20	<LOQ	0.39	0.32	0.19	0.35	0.05	0.69	3.22	This study
	Rio Doce estuary	Liver	0.51	0.19	0.42	2.36	0.11	7.20	0.05	2.14	27.27	This study
*Diapterus rhombeus*	Todos os Santos Bay (Polluted)	Muscle	<LOD	0.22	4.59	<LOD		0.61	<LOD		6.74	Santana et al. (2017)
	Morrão River estuary (Polluted)	Muscle		0.002		0.79			0.80		25.2	Bosco-Santos & Luiz-Silva (2019)
	Morrão River estuary (Polluted)	Liver		0.25		10.9			0.34		138	Bosco-Santos & Luiz-Silva (2019)
	Rio Doce estuary	Muscle	0.52	<LOQ	0.21	0.16	0.08	0.30	0.02	0.78	3.81	This study
	Rio Doce estuary	Liver	0.97	0.05	0.34	1.67	0.10	9.01	0.06	1.26	32.9	This study
*Mugil liza*	Itaipu - Guanabara Bay (Near-pristine)	Muscle		<LOQ		<LOQ		1.18			3.57	Hauser-Davis et al. (2016)
	Itaipu - Guanabara Bay (Near-pristine)	Liver		0.13		0.81		2.43			64.61	Hauser-Davis et al. (2016)
	Ipiranga - Guanabara Bay (Polluted)	Muscle		<LOQ		<LOQ		<LOQ			<LOQ	Hauser-Davis et al. (2016)
	Ipiranga - Guanabara Bay (Polluted)	Liver		0.06		<LOQ		0.59			74.31	Hauser-Davis et al. (2016)
	Morrão River estuary (Polluted)	Muscle		0.001		0.79			0.06		11.8	Bosco-Santos & Luiz-Silva (2019)
	Morrão River estuary (Polluted)	Liver		0.26		176			1.13		188	Bosco-Santos & Luiz-Silva (2019)
*Mugil curema*	Itaguaré River (Pristine)	Muscle			<LOD	0.27		0.51			6.68	Carmo, Abessa & Machado-Neto (2012)
	São Vicente estuary (Polluted)	Muscle			1.25	0.01		0.35			7.33	Carmo, Abessa & Machado-Neto (2012)
	São Vicente estuary (Polluted)	Muscle			1.70	0.02		0.25			6.75	Carmo, Abessa & Machado-Neto (2012)
*Mugil platanus*	Babitonga Bay (Polluted)	Muscle			0.06						4.40	Bonatti et al. (2004)
*Mugil sp*.	Vitória Bay (Polluted)	Muscle		0.030	0.151	0.214			0.27		3.26	Joyeux, Campanha Filho & de Jesus (2004)
	Rio Doce estuary	Muscle	0.25	<LOQ	0.32	0.30	0.07	0.20	<LOQ	0.50	3.21	This study
	Rio Doce estuary	Liver	1.73	0.11	0.46	47.30	0.16	1.85	0.04	5.81	71.71	This study

Trace metal concentrations varied between fish species and between liver and muscle tissues, probably as a result of varied physiological responses and exposure to contaminants (Shah & Altindağ, 2005). The inter- and intra -specific differences in protein expression suggest that fish sampled were either exposed to variable contaminant levels or that some fish species or individuals are less adapted to these stress levels. This hypothesis requires further investigation in the Rio Doce estuary. The fishes sampled in the Rio Doce estuary display demersal behavior, feeding on benthic invertebrates and other food sources, enabling possible direct ingestion of contaminated sediments and other pollutants (Dantas et al., 2019; Andrades et al., 2020). In addition, fish behavior may increase exposure to contaminants in sediments during active search for food on the bottom, leading to resuspension of contaminants and their intake through gills (Cline, 1994; Bustamante et al., 2003). The demersal catfish species of this region deserve special attention as they showed the highest trace metal concentration in both liver and muscle tissues and may increase human health risks when consumed. Differences in trace metal concentrations among species can also be associated to age, differences in metabolism or the presence of migratory behavior (Rodrigues et al., 2010). Fish age, in particular, may also reflect exposure periods in the environment and consequently influence bioaccumulation. Although we did not sample separate fish cohorts in our study, trace metal contamination and population patterns deserve future consideration as they could support decisions on fish consumption by vulnerable populations affected by the disaster.

This is, to the best of our knowledge, the first report on MT and GSH associations with metals in these fish species, except for *Mugil* sp., with a potential protective role against toxic elements. Their assessment could be an effective tool in the evaluation of metal contamination, once the expression of detoxifying biomarkers indicates current exposure to trace metals in aquatic ecosystems. Based on high trace metal contents in fish muscle, the consumption of demersal fish species poses risks to human health and should be prohibited in this estuarine region. In addition, estuarine health is probably compromised by chronic contamination, likely to be sub-optimal for fish development and fisheries production, both of which are important indirect effects neglected in management actions.

## Conclusions

Significant tissue bioaccumulation and oxidative stress defenses in fish were observed in response to contamination of the Rio Doce estuary by iron mine tailings. High concentrations of potentially toxic trace metals in the liver of the demersal species *G. genidens* and *C. spixii*, and their respective protein synthesis correlations, indicate chronic sublethal effects, while higher metallothionein levels in muscle tissues suggests metal overload in excretion organs. Trace metal concentrations in both liver and muscle tissue were above Brazilian and international guidelines for Maximum Residue Limits in foods for As, Cd, Cr, Cu, Mn, Pb and Zn, indicating potentially high human risks if consumed by communities near the impacted areas. Although our study evaluated these effects nearly 2 years after the disaster, these effects are likely to continue as long as the tailings are still being deposited in the estuarine ecosystem, which will also likely offer sub-optimal conditions for the development of fish species. Our study supports the use of demersal fish species used in the present study in an environmental biomonitoring context, which may improve the performance of current and future short and long-term impact assessment studies.

## Supplemental Information

10.7717/peerj.10266/supp-1 Supplemental Information 1 Biometric data regarding the sampled fish from Rio Doce estuary in August 2017. Data is displayed as means ± SD.

10.7717/peerj.10266/supp-2 Supplemental Information 2 Limits of detection and quality control of total element content in sediments determined by the USEPA 3052 method. Certified reference material: NIST SRM 2709a.

10.7717/peerj.10266/supp-3 Supplemental Information 3 Observed and certified values (mg kg^−1^) for the DORM-4 certified reference material, the recoveries (%) for each determined element, and their respective LOQ and LOD (mg kg^−1^) for fish tissues.

10.7717/peerj.10266/supp-4 Supplemental Information 4 Raw data of metal concentrations in sediments sampled in the Rio Doce estuary in August 2017.

10.7717/peerj.10266/supp-5 Supplemental Information 5 Raw data of metals and oxidative stress biomarkers in liver and muscle tissues of *C. spixii* (*n* = 15), *E. brasilianus* (*n* = 18), *Mugil* sp. (*n* = 11) and *D. rhombeus* (*n* = 9) sampled in the Rio Doce estuary in August 2017.

10.7717/peerj.10266/supp-6 Supplemental Information 6 Raw data of environmental surface water parameters collected in August 2017 in the Rio Doce estuary. TDS - total dissolved solids.

## Additional Information and Declarations

### Field Study Permissions

The following information was supplied relating to field study approvals (i.e., approving body and any reference numbers): The field sampling was conducted under SISBIO/ICMBio license number 57819-4.

### Data Availability

The following information was supplied regarding data availability: The raw measurements are available in the Supplemental Files.
