# Supplementary material for: Contamination and oxidative stress biomarkers in estuarine fish following a mine tailing disaster
Source: PeerJ. 2020 Oct 28;8:e10266. doi: 10.7717/peerj.10266 (PMC7602685; doi:10.7717/peerj.10266)
Supplement: Supplemental Information 1 — Data is displayed as means ± SD. [file peerj-08-10266-s001.docx]

SUPPLEMENTARY MATERIAL

**Contamination and oxidative stress biomarkers in estuarine fish following a mine tailing disaster.**

Table S1. Biometric data regarding the sampled fish from Rio Doce estuary in August 2017. Data is displayed as means ± SD.

|  | *C. spixii*  N=15 | *G. genidens*  N=18 | *E. brasilianus*  N=18 | *D. rhombeus*  N=9 | *Mugil* sp.  N=11 |
| --- | --- | --- | --- | --- | --- |
| Length (cm)  (min-max) | 30.7 ± 7.2  (20.7 – 44.5) | 24.3 ± 4.2  (19.8 – 33.8) | 27.5 ± 5.1  (20.7 – 34.0) | 17.9 ± 4.6  (15.0 – 27.0) | 44.2 ± 8.9  (20.9 – 55.0) |
|  |  |  |  |  |  |
| Weight (g)  (min-max) | 246.0 ± 113.1  (98.6 – 445.2) | 156.2 ± 78.3  (72.5 – 223.9) | 206.9 ± 98.8  (103.4 – 369.8) | 101.3 ± 52.2  (86.8 – 188.4) | 515.6 ± 223.3  (142.3 – 950.2) |
