# Supplementary material for: Contamination and oxidative stress biomarkers in estuarine fish following a mine tailing disaster
Source: PeerJ. 2020 Oct 28;8:e10266. doi: 10.7717/peerj.10266 (PMC7602685; doi:10.7717/peerj.10266)
Supplement: Supplemental Information 2 — Certified reference material: NIST SRM 2709a. [file peerj-08-10266-s002.docx]

SUPPLEMENTARY MATERIAL

**Contamination and oxidative stress biomarkers in estuarine fish following a mine tailing disaster.**

Table S2. Limits of detection and quality control of total element content in sediments determined by the USEPA 3052 method. Certified reference material: NIST SRM 2709a.

| Quality assurance | As | Cd | Cr | Cu | Pb | Zn |
| --- | --- | --- | --- | --- | --- | --- |
| Detection limit | 0.01 | 0.01 | 0.01 | 0.01 | 0.01 | 0.01 |
| Measured value | 1.068 | 0.9619 | 0.9608 | 1.012 | 0.9917 | 0.9989 |
| Certified value | 1 | 1 | 1 | 1 | 1 | 1 |
| Recovery (%) | 106.8 | 96.19 | 96.08 | 101.2 | 99.17 | 99.89 |
