# Supplementary material for: Contamination and oxidative stress biomarkers in estuarine fish following a mine tailing disaster
Source: PeerJ. 2020 Oct 28;8:e10266. doi: 10.7717/peerj.10266 (PMC7602685; doi:10.7717/peerj.10266)
Supplement: Supplemental Information 3 [file peerj-08-10266-s003.docx]

SUPPLEMENTARY MATERIAL

**Contamination and oxidative stress biomarkers in estuarine fish following a mine tailing disaster.**

Table S3. Observed and certified values (mg kg^-1^) for the DORM-4 certified reference material, the recoveries (%) for each determined element, and their respective LOQ and LOD (mg kg^-1^) for fish tissues.

| Element | Observed value | Certified value | Recovery (%) | LOQ | LOD |
| --- | --- | --- | --- | --- | --- |
| As | 6.41 ± 0.26 | 6.87 ± 0.44 | 93.30 | 0.0943 | 0.0283 |
| Cd | 0.28 ± 0.07 | 0.299 ± 0.018 | 93.64 | 0.0255 | 0.0077 |
| Cr | 2.22 ± 0.29 | 1.87 ± 0.18 | 118.71 | 0.2433 | 0.0730 |
| Cu | 12.59 ± 0.71 | 15.7 ± 0.46 | 80.19 | 0.0200 | 0.0060 |
| Mn | 2.83 ± 0.71 | - | - | 0.0067 | 0.0020 |
| Hg | 0.33 ± 0.06 | 0.412 ± 0.0036 | 80.09 | 0.0254 | 0.0076 |
| Pb | 0.40 ± 0.13 | 0.404 ± 0.062 | 99.00 | 0.0126 | 0.0038 |
| Se | 3.63 ± 0.25 | 3.45 ± 0.40 | 105.21 | 0.0851 | 0.0255 |
| Zn | 41.49 ± 1.10 | 51.6 ± 2.8 | 80.40 | 0.1516 | 0.0455 |
